# MICA*019 Allele and Soluble MICA as Biomarkers for Ankylosing Spondylitis in Taiwanese

**DOI:** 10.3390/jpm11060564

**Published:** 2021-06-16

**Authors:** Chin-Man Wang, Keng-Poo Tan, Yeong-Jian Jan Wu, Jing-Chi Lin, Jian-Wen Zheng, Alice L. Yu, Jian-Ming Wu, Ji-Yih Chen

**Affiliations:** 1 Department of Rehabilitation, Chang Gung Memorial Hospital, Chang Gung University College of Medicine, Taoyuan 33302, Taiwan; cmw1314@adm.cgmh.org.tw; 2 Department of Medicine, Division of Allergy, Immunology and Rheumatology, Chang Gung Memorial Hospital, Chang Gung University College of Medicine, Taoyuan 33302, Taiwan; helentan.tw@yahoo.com.tw (K.-P.T.); yjwu1962@gmail.com (Y-.J.J.W.); jingchilin@gmail.com (J.-C.L.); pqr780925@cgmh.org.tw (J.-W.Z.); 3 Institute of Stem Cell and Translational Cancer Research, Chang Gung Memorial Hospital at Linkou, Taoyuan 33375, Taiwan; a1yu@ucsd.edu; 4 Department of Pediatrics, University of California, San Diego, CA 92103, USA; 5 Department of Veterinary and Biomedical Sciences, Department of Medicine, University of Minnesota, Minneapolis, MN 55108, USA; jmwu@umn.edu

**Keywords:** MICA, AS, soluble MICA, HLA-B27, syndesmophyte

## Abstract

MICA (major histocompatibility complex class I chain-related gene A) interacts with NKG2D on immune cells to regulate host immune responses. We aimed to determine whether *MICA* alleles are associated with AS susceptibility in Taiwanese. *MICA* alleles were determined through haplotype analyses of major *MICA* coding SNP (cSNP) data from 895 AS patients and 896 normal healthy controls in Taiwan. The distributions of *MICA* alleles were compared between AS patients and normal healthy controls and among AS patients, stratified by clinical characteristics. ELISA was used to determine soluble MICA (sMICA) levels in serum of AS patients and healthy controls. Stable cell lines expressing four major *MICA* alleles (MICA*002, MICA*008, MICA*010 and MICA*019) in Taiwanese were used for biological analyses. We found that MICA*019 is the only major *MICA* allele significantly associated with AS susceptibility (P*_FDR_* = 2.25 × 10^−115^; OR, 14.90; 95% CI, 11.83–18.77) in Taiwanese. In addition, the MICA*019 allele is associated with syndesmophyte formation (P*_FDR_* = 0.0017; OR, 1.69; 95% CI, 1.29–2.22) and *HLA-B27* positivity (P*_FDR_* = 1.45 × 10^−33^; OR, 28.79; 95% CI, 16.83–49.26) in AS patients. Serum sMICA levels were significantly increased in AS patients as compared to healthy controls. Additionally, MICA*019 homozygous subjects produced the highest levels of sMICA, compared to donors with other genotypes. Furthermore, in vitro experiments revealed that cells expressing MICA*019 produced the highest level of sMICA, as compared to other major *MICA* alleles. In summary, the MICA*019 allele, producing the highest levels of sMICA, is a significant risk factor for AS and syndesmophyte formation in Taiwanese. Our data indicate that a high level of sMICA is a biomarker for AS.

## 1. Introduction

Ankylosing spondylitis (AS), a form of insidious and debilitating spondyloarthritis (SpA), is characterized by chronic inflammation and osteo-proliferation of the axial skeletons, including the spine and sacroiliac joints, usually resulting in bone fusion of affected areas [1]. Cells of innate and adaptive immune systems participate in the initiation, development and perpetuation of AS. However, the most relevant immune cell type in the pathogenesis of AS has not been fully elucidated [2]. Natural killer (NK) cells, particularly the CD56^bright^ subset of NK cells with immune-regulatory properties, accumulate in inflammatory tissue sites (such as synovial membrane and skin lesions) of rheumatoid arthritis and psoriatic patients [3,4,5,6]. AS patients have increased frequencies of CD56^+^CD16^+^ and CD56^dim^CD16^+^ NK cells, indicating a role of NK cells or subsets of NK cells in AS pathogenesis [7,8,9].

NK cells, a type of lymphocytes of the innate immune system, serve as regulatory immune cells in shaping adaptive immune responses by interacting with dendritic cells, macrophages, T cells and endothelial cells. NK cells represent the founding family member of the innate lymphoid cells (ILCs), which functionally inhibit or exacerbate immune responses based on signals from inhibitory and activating receptors [10,11]. Accumulating evidence points to the concept that NK cells are effector cells that can either contribute to or protect against inflammation and autoimmunity [12,13,14,15,16]. NK cells play a protective role in the development of experimental arthritis, an effect possibly mediated by suppressing Th17 cell generation via IFN-γ production [17].

The breakdown of the delicate balance between immune activation and tolerance leads to autoimmune responses. The major histocompatibility complex class I chain-related gene A (MICA) regulates immune responses through interaction with NKG2D (receptor natural killer group 2, member D), which mediates activation or co-stimulation of NK cells and subsets of T cells [18,19,20]. MICA, a member of non-classical MHC class I family, is highly polymorphic, reminiscent of the classical MHC class I genes. Numerous *MICA* alleles exist in human populations. Previous genetic and functional analyses of *MICA* variants shed some light into mechanistic roles of MICA in immune responses and inflammation [21,22,23,24]. *MICA* coding SNP (cSNPs) and alleles were associated with AS susceptibility in American Caucasians and Han Chinese populations [25]. Additionally, *MICA* promoter SNP rs4349859, that was previously used as the *HLA-B27*-tag SNP, had the strongest association with AS in Europeans [26]. However, the *MICA* allele profiles vary significantly in different ethnic populations. Different genetic backgrounds and environmental factors may also influence the effect of *MICA* alleles on the pathogenesis of inflammatory diseases. Therefore, *MICA* allele or alleles may affect AS susceptibility in a population-specific fashion. Additionally, phenotype–genotype analyses of population-specific *MICA* alleles may provide insights into the mechanistic role of *MICA* alleles in the pathogeneses of AS. In the current study, we established a comprehensive profile of *MICA* alleles in Taiwanese by sequence analyses of a large number of human subjects. Most importantly, we identified the MICA*019 allele as a major risk factor for AS in Taiwanese. Ex vivo experiments revealed that genotypes containing the MICA*019 allele are significantly associated with serum soluble MICA (sMICA) levels. Our data suggest a unique role for MICA*019 in the development of AS in Taiwanese.

## 2. Materials and Methods

### 2.1. Study Subjects

A total of 895 AS patients (725 males and 170 females), who fulfilled the 1984 revised New York diagnostic criteria for AS [27], were recruited at the Chang Gung Memorial Hospital in Taiwan. Two rheumatologists independently evaluated lateral syndesmophyte formation and graded the severity of AS, according to the modified Stoke Ankylosing Spondylitis Spinal Score (mSASSS), after a 10-year observation [28]. Based on radiography, patients were separated into group 1 (no syndesmophytes), group 2 (fewer than 4 syndesmophytes, mSASSS < 24), or group 3 (4 or more syndesmophytes, mSASSS ≥ 24), as previously described [29]. Detailed demographical information of the study subjects is listed in Supplementary Table S1. A total of 896 healthy blood donors (752 males and 144 females) were recruited as normal healthy controls. One husband and twelve AS patients were tested for sMICA levels who received NSAID and DMARDs (sulfasalazine mostly) but not biologic agents. All experimental protocols were approved by the ethics committee of Chang Gung Memorial Hospital with IRB protocol# 104-9983B and informed consents were obtained from all subjects.

### 2.2. DNA Sequence Analysis of MICA

The *MICA*-specific genomic DNA fragment containing exons 2, 3, 4 and 5 amplified by PCR was used for DNA sequence analysis of *MICA*. The IMGT/HLA database (http://ftp.ebi.ac.uk/pub/databases/ipd/imgt/hla/fasta/MICA_nuc.fasta, release date: 2020 (accessed on 1 January 2021)) was used to assign 105 *MICA* haplotypes or alleles.

### 2.3. Determination of Soluble MICA (sMICA) Levels in Serum Samples

Serum samples of normal healthy controls and AS patients were used in the ELISA analysis of sMICA. Serum sMICA levels were determined in triplicates using a sandwich MICA DuoSet ELISA kit (R&D Systems, Minneapolis, MN, USA), according to the manufacturer’s instructions.

### 2.4. Generation of Human MICA Expression Constructs

*MICA* cDNAs of peripheral blood mixed mononuclear cells from the carriers of *MICA* alleles (MICA*002, MICA*008, MICA*010 and MICA*019) were amplified by RT-PCR and were subsequently cloned into the lentiviral vector pCDH-CMV-EF1-copGFP (Systems Biosciences, Palo Alto, CA, USA).

### 2.5. Generation of Cell Lines Expressing MICA Alleles

The C1R (ATCC#CRL-1573, Manassas, VA, USA) and LCL-721.221 (ATCC#CRL-1855) stable cell lines expressing empty vector MICA*002, MICA*008, MICA*010, or MICA*019 alleles were established for analyses of sMICA, exosomal MICA and cellular MICA. Membrane-bound MICA was analyzed by the flow cytometry analysis.

### 2.6. Western Blot Analyses and Detection of sMICA, Exosomal MICA and Cellular MICA

The ultracentrifugation supernatant fractions of cell culture medium containing sMICA were immunoprecipitated with anti-human MICA/MICA (Biolegend, San Diego, CA, USA) and protein G Mag sepharose Xtra beads (GE Healthcare, Chicago, IL, USA). The immunoprecipitation-concentrated sMICA, the isolated exosomes and total cell lysate were used for western blot analyses to determine amounts of sMICA, exosomal MICA and cellular MICA.

### 2.7. Statistical Analysis

Single-locus analyses of *MICA* cSNPs were performed to compare distributions of genotypes and alleles between normal healthy controls and AS patients. Linkage disequilibrium (LD) between marker loci or cSNPs was measured and haplotype blocks were constructed using Haploview 4.2 (Broad Institute, Cambridge, MA, USA; http://www.broad.mit.edu/mpg/haploview (accessed on 1 October 2020)). Associations of the estimated haplotypes or alleles and disease status were tested in logistic regression models. The 5% level of significance for *p*-values was used for all the analyses.

### 2.8. Supplementary Methods

Detailed materials and methods are described in the Supplementary Methods section of the Online Supplementary Information.

## 3. Results

### 3.1. MICA cSNP and Alleles in Taiwanese

The spectra of *MICA* cSNPs and alleles were determined by sequencing analysis of 896 Taiwanese healthy blood donors (normal healthy controls) and 895 AS patients. Among 32 total *MICA* cSNPs, that were identified in the combined population of healthy controls and AS patients, 22 are common cSNPs within MICA extracellular domains (seven in exon 2: rs9380254, rs1063630, rs1063631, rs1051785, rs17200158, rs1051786 and rs1063632; eight in exon 3: rs1051790, rs41539919, rs1051792, rs576467210, rs3819268, rs1051794, rs1131896 and rs1131897; seven in exon 4: rs17206680, rs1051796, rs1051797, rs1051798, rs1140700, rs1051799 and rs1063635). Sixteen common cSNPs (four in the exon 2, seven in the exon 3 and five in the exon 4) are non-synonymous cSNPs that lead to changes of amino acid codons in the MICA extracellular domains (Supplementary Table S2). In the combined population of normal controls and AS patients, the total number of *MICA* alleles was estimated to be >100, among which nine *MICA* alleles (MICA*019, MICA*008, MICA*010, MICA*002, MICA*004, MICA*012, MICA*045,MICA*033 and MICA*007) have the allele frequencies of >0.01 (Table 1). The linkage disequilibrium (LD) analysis confirmed that several *MICA* allele-tag SNPs are in strong LD (r^2^ > 0.9) with specific *MICA* alleles in Taiwanese (Supplementary Table S3).

### 3.2. Association of MICA cSNPs with AS Susceptibility in Taiwanese

Single-locus analyses revealed that most of *MICA* cSNPs were significantly associated with AS susceptibility and *HLA-B27* positivity in AS patients (Supplementary Tables S4 and S5).

### 3.3. Association of MICA Alleles with AS Susceptibility in Taiwanese

In normal healthy controls, only seven *MICA* alleles (MICA*019, MICA*008, MICA*010, MICA*002, MICA*004, MICA*012 and MICA*045) had allele frequencies of >0.03 (or >3%). We assigned those seven *MICA* alleles as Taiwanese-specific major alleles, which account for about 93% of total *MICA* alleles identified in Taiwanese normal healthy population (Table 1). As shown in Table 1, MICA*019 is the only main allele that was significantly associated with a risk for AS susceptibility (P*_FDR_* = 2.25 × 10^−115^; OR, 14.90; 95% CI, 11.83–18.77). On the other hand, five main *MICA* alleles (MICA*008, MICA *010, MICA *002, MICA *004 and MICA *012) were significantly associated with protection against AS (MICA*008: P*_FDR_* = 8.98 × 10^−12^; OR, 0.56; 95% CI, 0.48–0.66. MICA*010: P*_FDR_* = 4.5 × 10^−6^; OR, 0.61; 95% CI, 0.50–0.75. MICA*002: P*_FDR_* = 7.33 × 10^−16^; OR, 0.44; 95% CI, 0.37–0.54. MICA*004: P*_FDR_* = 4.98 × 10^−5^; OR, 0.54 95% CI, 0.40–0.72. MICA*012: P*_FDR_* = 2.11 × 10^−9^; OR, 0.37; 95% CI, 0.27–0.51).

### 3.4. Associations of MICA Alleles with Syndesmophyte Formation in AS Patients

Syndesmophytes reflect main features of spinal structural damage of AS, due to inflammation and ossification of the outer fibers of the annulus fibrosus. Among seven main *MICA* alleles, MICA*019 was significantly associated with the risk for syndesmophyte formation (P*_FDR_* = 0.0017; OR, 1.69; 95% CI, 1.29–2.22) among AS patients (Table 2).

### 3.5. Associations of MICA Alleles with HLA-B27 Positivity in AS Patients

*HLA-B27* is a recognized genetic risk factor for AS. In the current study, we found that MICA*019 was also significantly associated with *HLA-B27* positivity (P*_FDR_* = 1.45 × 10^−33^; OR, 28.79; 95% CI, 16.83–49.26) in AS patients (Table 3). On the other hand, five out of seven main *MICA* alleles were negatively associated with *HLA-B27* positivity in AS patients. Our data suggest that MICA*019 allele may genetically interact with *HLA-B27* to contribute to the development of AS. To control the effect of linkage disequilibrium between *MICA* alleles and *HLA-B27* on AS, we carried out an *MICA* genetic analysis of AS patients and healthy controls stratified by the presence and absence of *HLA-B27*. As shown in Supplementary Table S6, MICA*019 resulted to significantly associate with AS susceptibility (P*_FDR_* = 0.012; OR, 2.84; 95% CI, 1.51–5.33; power: 0.581) in analyzing *HLA-B27* positive AS patients and *HLA-B27* positive healthy controls, indicating that the MICA*019 allele is an additional AS risk factor besides *HLA-B27*. Nevertheless, MICA*019 was not significantly associated with AS susceptibility among subjects negative for *HLA-B27* (*p* = 0.925; OR, 1.11; 95% CI, 0.67–1.86; power: 0.083) (Supplementary Table S7). Fitted logistic regression models controlling for sex and age were used to test the interactions of HLA-B27 and MICA alleles (one at a time). We found that the interaction between HLA-B27 and MICA*019 had a significant effect on AS (*p* = 0.0241), while all other interactions were not significant (*p* > 0.05). Taken together, our data suggest that MICA*019 and *HLA-B27* may play synergistic roles in the pathogenesis of AS.

### 3.6. Increased Levels of Serum sMICA in AS Patients and MICA*019 Homozygous AS Patients

**Figure 1:**
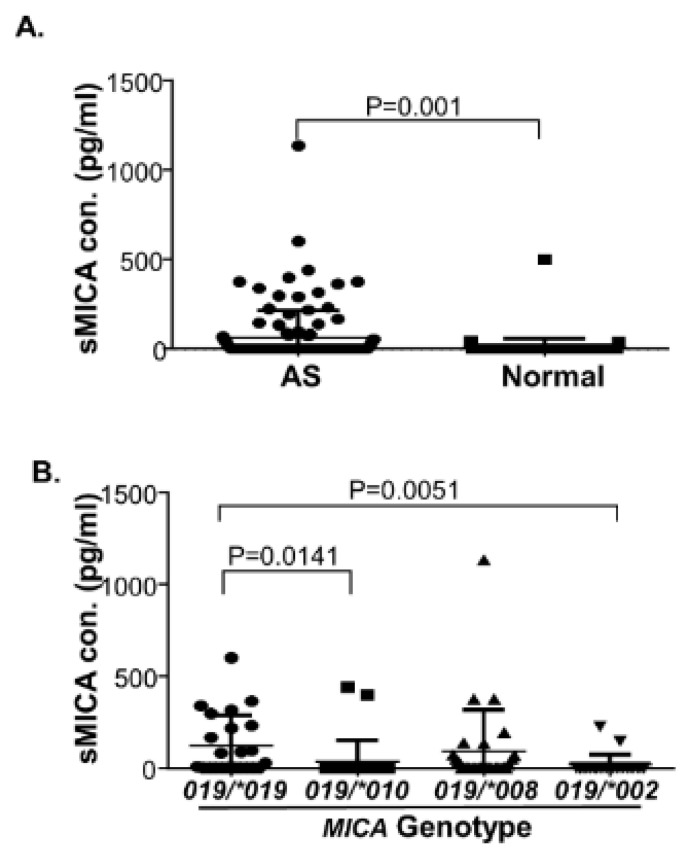
Elevated levels of soluble MICA (sMICA) in AS patients and MICA*019 homozygous genotype subjects. (**A**) Serum sMICA levels in AS patients and normal healthy controls. The serum sMICA levels were significantly increased in AS patients (N = 112, sMICA concentration: 63.35 ± 14.65 pg/mL), as compared to normal healthy controls (N = 92, sMICA concentration: 6.224 ± 5.417 pg/mL) (*p* = 0.001). (**B**) Serum sMICA levels in AS patients carrying MICA*019 allele. MICA*019/*019 subjects produced significantly more sMICA (N = 23, sMICA concentration: 122.8 ± 34.39 pg/mL) than MICA*019/*010 (N = 30, sMICA concentration: 27.88 ± 19.40) (*p* = 0.0141) and MICA*019/*002 (N = 23, sMICA concentration: 16.10 ± 11.39) (*p* = 0.0051) subjects. MICA*019/*019 subjects produced higher levels of sMICA (N = 23, sMICA concentration: 122.8 ± 34.39 pg/mL) than MICA*019/*008 subjects (N = 29, sMICA concentration: 90.95 ± 41.76 pg/mL), but the difference was statistically insignificant.

To examine the role of MICA in AS, we determined sMICA levels in AS patients and normal healthy controls. As shown in Figure 1A, we found that serum sMICA concentrations were significantly increased in AS patients (N = 112, sMICA concentration: 63.35 ± 14.65 pg/mL), as compared to normal healthy controls (N = 92, sMICA concentration: 6.224 ± 5.417 pg/mL) (*p* = 0.001). Since MICA*019 is the only main allele that was significantly associated with AS susceptibility, sMICA concentrations of AS patients who were either homozygous or heterozygous for MICA*019 were used to analyze the effects of different *MICA* alleles on sMICA levels in the presence of MICA*019. As shown in Figure 1B, MICA*019 homozygous subjects (MICA*019/*019) produced significantly higher levels of sMICA than subjects with MICA*019/*010 (*p* = 0.0141) and MICA*019/*002 (*p* = 0.0051) genotypes. Although MICA*019 homozygous subjects (MICA*019/*019) produced higher levels of sMICA than MICA*019/*008 heterozygous subjects, the difference did not reach statistical significance. Our data indicate that MICA*019 significantly affects sMICA production. Notably, no single SNP was found to associate with sMICA levels and serum sMICA concentrations were not correlated with ESR, CRP, BASFI (Bath Ankylosing Spondylitis Functional Index) and BASDAI (Bath Ankylosing Spondylitis Disease Activity Index) (data not shown).

### 3.7. Cells Expressing MICA*019 Allele Produces the Highest Amount of sMICA In Vitro

**Figure 2:**
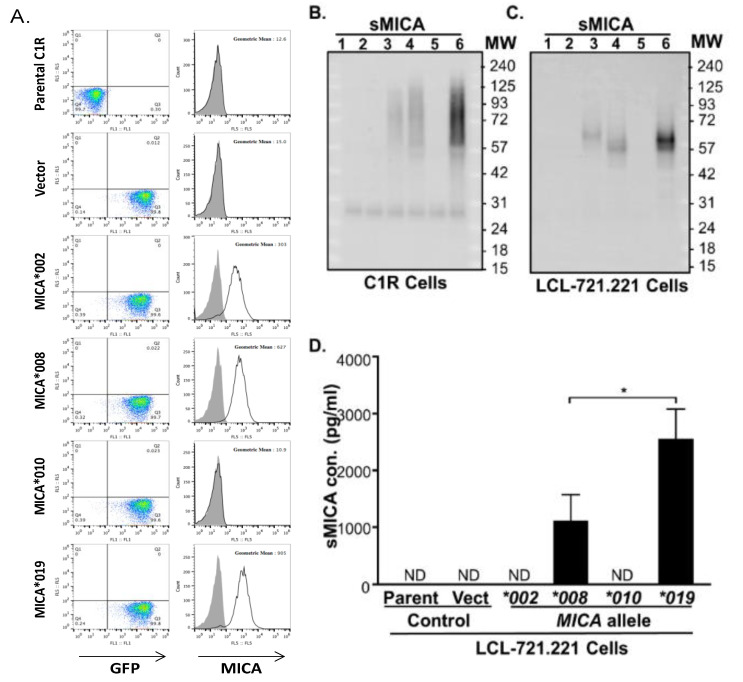
Surface expressions and soluble MICA (sMICA) productions of *MICA* alleles. (**A**) Representatives of transduced cells expressing vector and *MICA* alleles (MICA*002, MICA*008, MICA*010 and MICA*019) were sorted for equivalent GFP expressions (left panels). The MICA*019 cells expressed the highest level of surface MICA (geometric mean: 905), as compared to MICA*002 (geometric mean: 303) and MICA*008 (geometric mean: 627) cells, while MICA*010 cells failed to express surface MICA on cell membrane. (**B**) Western blot analysis of sMICA produced by C1R cells expressing *MICA* alleles. Ultracentrifugation was used to separate cell-free culture supernatants into supernatant fractions containing sMICA. Immunoprecipitation-concentrated sMICA were subjected to western blot analysis, as described in Materials and Methods. No sMICA could be detected from cell culture supernatants of parental C1R cells (Lane 1) and C1R cells expressing vector control (Lane 2). C1R cells expressing MICA*019 (Lane 6) produced the most sMICA in culture supernatants among cells expressing MICA*002 (Lane 3), MICA*008 (Lane 4), MICA*010 (Lane 5) and MICA*019. MICA*008 cells (Lane 4) produced more sMICA than MICA*002 cells (Lane 3), while MICA*010 cells (Lane 5) produced little if any sMICA. (**C**) Western blot analysis of culture supernatants from parental LCL-721.221 cells (Lane 1) and LCL-721.221cells stably expressing vector control (Lane 2), MICA*002 (Lane 3), MICA*008 (Lane 4), MICA*010 (Lane 5) and MICA*019 (Lane 6) alleles. Similar results were obtained as in C1R cells. (**D**) ELISA analysis of sMICA in the culture supernatants of the LCL-721.221 cells stably expressing *MICA* allele. The MICA*019 cell culture supernatant contained the highest level of sMICA, followed by the MICA*008 cell culture supernatant. There were no detectable levels (ND) of sMICA in cell culture supernatants from parental LCL cells (parent), cells expressing empty vector (vector) and MICA*002 and MICA*010 alleles. All experiments were repeated at least three times and representative data are shown.

**Figure 3:**
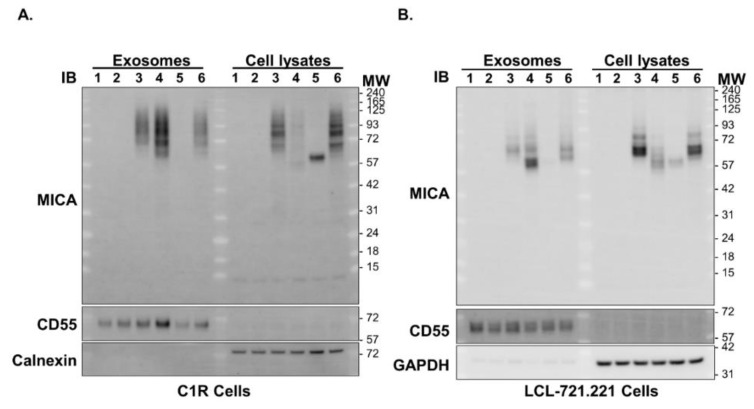
Western blot analyses of MICA from exosomes and cell lysates. (**A**) Ultracentrifugation of culture supernatants of C1R parental cells (Lane 1), C1R cells stably expressing empty vector (Lane 2), MICA*002 (Lane 3), MICA*008 (Lane 4), MICA*010 (Lane 5) and MICA*019 (Lane 6) was used to obtain exosomes in ultracentrifugation pellets. The parental C1R cells (Lane 1), C1R cells stably expressing empty vector (Lane 2), MICA*002 (Lane 3), MICA*008 (Lane 4), MICA*010 (Lane 5) and MICA*019 (Lane 6) were lysed in lysis buffer to generate total cell lysates. Exosomes and total cell lysates were subjected to western blot analysis, as described in Materials and Methods. CD55 was used as a loading control to normalize the expression of MICA in exosomes and GAPDH as a loading control to normalize the expression of MICA in total cell lysates. C1R cells expressing MICA*008 (Lane 4) produced the highest amount of exosomal MICA compared to cells expressing MICA*002 (Lane 3) and MICA*019 (Lane 6), while no exosomal MICA was produced from MICA*010 (Lane 5) cells. Lysates of MICA*002 (Lane 3) and MICA*019 (Lane 6) cells contained more intracellular MICA than MICA*008 (Lane 4) and MICA*010 (Lane 5) cells. (**B**) Western blot analyses of exosomes and cell lysates from parental LCL-721.221 cells (Lane 1) and LCL-721.221cells stably expressing vector control (Lane 2), MICA*002 (Lane 3), MICA*008 (Lane 4), MICA*010 (Lane 5) and MICA*019 (Lane 6) alleles. Similar results were obtained as in C1R cells. All experiments were repeated at least three times and representative data are shown.

MICA*019 is significantly associated with susceptibility to AS disease, while MICA*002, MICA*008 and MICA*010 are protective against AS development (Table 1). In addition, MICA*019 homozygosity is associated with increased sMICA concentrations, whereas the presence of the MICA*002, MICA*008 and MICA*010 alleles is associated with lower levels of sMICA in AS patients (Figure 1B). To confirm the effect of MICA*019 on sMICA production, we carried out in vitro experiments using C1R and LCL-721.221 cell lines stably expressing MICA*019, MICA*002, MICA*008, or MICA*010 alleles. As shown in Figure 2A (right column of panels), MICA*019 cells expressed the highest level of surface MICA (geometric mean: 905), as compared to MICA*002 (geometric mean: 303) and MICA*008 (geometric mean: 627) cells. However, MICA*010 cells failed to express surface MICA on cell membrane, which is consistent with previous findings by Li et al. [30]. The immunoblot analysis revealed that C1R cells expressing MICA*019 cells (Lane 6) produced the highest amount of sMICA in culture supernatants among cells expressing four *MICA* alleles (MICA*002, MICA*008, MICA*010 and MICA*019) (Figure 2B). MICA*008 cells (Lane 4) produced more sMICA than MICA*002 cells (Lane 3), while MICA*010 cells (Lane 5) produced little if any sMICA in western blot analyses. Similar results were obtained with LCL-721.221 cells stably expressing MICA*002, MICA*008, MICA*010 and MICA*019 alleles (Figure 2C). We also carried out ELISA to determine sMICA levels in the culture supernatants of the LCL-721.221 cells stably expressing MICA*002, MICA*008, MICA*010 or MICA*019. Again, the MICA*019 cell culture supernatant contained the highest level of sMICA, which was followed by the MICA*008 cell culture supernatant (Figure 2D). Surprisingly, sMICA concentrations were so low in the MICA*002 and MICA*010 cell culture supernatants that sMICA were undetectable by ELISA (Figure 2D). Our data confirmed that the MICA*019 allele is the highest sMICA producer, while MICA*008 also produces significantly more sMICA than MICA*002 and MICA*010, suggesting a biological mechanism by which homozygous MICA*019/019 subjects produced the highest levels of sMICA among subjects carrying at least one MICA*019 allele (Figure 1B). Interestingly, we found that MICA*008 cells (Lane 4) produced the highest amount of exosomal MICA among four *MICA* alleles (Figure 3A,B). Although the MICA protein could be detected in MICA*010 cell lysates (Lane 5, right panels of Figure 3A,B) in both C1R and LCL cells, MICA*010 cells failed to expressed cell surface MICA (Figure 2A). In addition, MICA*010 was unable to produce detectable amounts of sMICA (Figure 2B–D) and exosomal MICA production (Lane 5, left panels of Figure 3A,B), suggesting the MICA*10 protein is intracellularly trapped and is unable to express as surface MICA and sMICA.

## 4. Discussion

MHC gene composition (especially the positivity of *HLA-B27*) confers the greatest risk for AS, while non-MHC genes also contribute to the AS development process [31,32,33]. In this study, we have determined the comprehensive profile of *MICA* alleles in Taiwanese by analyzing a large number of subjects. We have obtained the most extensive *MICA* allele dataset that has ever been assembled for an Asian population. Most importantly, the MICA*019 allele was identified as a major risk factor for AS in Taiwanese. Phenotype–genotype analyses revealed that MICA*019 homozygosity was associated with the highest levels of sMICA production in Taiwanese AS patients. In vitro experiments confirmed that MICA*019 cells produced the larger amount of sMICA among main *MICA* alleles of Taiwanese. Our data suggest that *MICA* plays an important role in the development of AS, possibly through the increased production of sMICA.

MICA plays important roles in tumor surveillance and inflammation [22,34]. DNA damage, heat, viral infection and inflammation promote the expression of MICA, which triggers the activation of lymphocytes for immune responses. By engaging NKG2D and overriding the inhibitory signals of killer inhibitory receptors (KIRs) and/or CD94/NKG2A/B, MICA effectively activates NK, γδ T cells and αβ CD8^+^ T cells to kill virus-infected cells or tumor cells [34,35,36]. The *MICA* cSNP rs1051792G>A that causes amino acid substitution from valine to methionine at the position 129 (MICA-129Val>Met) significantly affects the interaction strength between MICA and NKG2D, the production of sMICA and the density of MICA on the plasma membrane [34,37,38,39]. The MICA-129Met variant has stronger binding affinity for NKG2D than MICA-129Val, leading to increased cytotoxicity and IFN-γ release from NK cells and CD8^+^ T cells [37,40]. The interaction between high affinity the MICA-129Met variant and NKG2D could effectively downregulate NKG2D cell surface expression, impacting clinical outcomes of GVHD [38]. The MICA-129Val/Val homozygous genotype has been associated with higher levels of sMICA and the progression of multiple myeloma [40]. The microsatellite triplet repeat polymorphism within transmembrane segment also affect MICA functions [34]. However, functions of the *MICA* cSNP rs1051792G>A were mostly analyzed without considering the effect of other *MICA* cSNPs that may modify the functions of the SNP rs1051792G>A. Multiple *MICA* alleles share the same SNP rs1051792G>A allele (e.g., both MICA*019 and MICA*004 contain the rs1051792G or 129Val allele; Supplementary Table S2), but have opposite effects on disease susceptibility (MICA*019 is a risk factor for AS, while MICA*004 is protective against AS; Table 1). Therefore, the associations of *MICA* cSNP rs1051792G>A (or MICA-129Val>Met) single locus with various disease susceptibilities need to be reassessed and *MICA* alleles (or *MICA* cSNP haplotypes) should be used to accurately evaluate the contribution of *MICA* to a specific disease in future association studies.

*MICA* alleles are associated with susceptibility to chronic autoimmune inflammatory diseases. The MICA*007 allele was reportedly associated with AS and ulcerative colitis (UC), while MICA*019 was associated with AS and Bechet’s disease [25,41,42]. The associations of *MICA* variations with AS were examined in cohorts of European Americans and Han Chinese, leading to the identification of MICA*007 as a significant risk allele for AS in both Caucasian and Han Chinese populations, independent of *HLA-B27* (US cohort OR = 60.66; Chinese cohort OR = 7.78) [25]. However, a recent study with a large cohort of European ancestry (9429 AS cases and 13,459 controls) demonstrated no evidence of association between the MICA*007 allele and AS susceptibility. The lack of association between the MICA*007 allele and AS risk was further confirmed in a relatively homogenous UK population (4198 AS cases, 9611 controls), which excluded the effect of population heterogeneity on the negative disease association between the MICA*007 allele and AS risk [43]. In the current study, we revealed a lack of association between MICA*007 and AS susceptibility in Taiwanese. Therefore, it is questionable that the MICA*007 allele is a common risk factor for AS in humans.

Besides MICA*007, MICA*019 was identified as another independent risk allele for AS in Han Chinese, while MICA*019 was not associated with AS susceptibility in Americans with European ancestry, indicating that MICA*019 may have a role in AS development in Han Chinese despite the proximity of *MICA* and *HLA-B27* [25]. We found that MICA*019 is the only major risk allele for AS in Taiwanese and that MICA*019 is also significantly associated with *HLA-B27* positivity in AS patients. MICA*019 resulted to significantly associate with AS susceptibility in comparing *HLA-B27* positive AS patients with *HLA-B27* positive healthy controls, indicating that the MICA*019 allele is an additional risk factor for AS. Moreover, MICA*019 is significantly associated with syndesmophyte formation in AS patients, suggesting that MICA*019 is also a biomarker for AS severity. Male sex, elevated serum C-reactive protein levels and preexisting syndesmophytes are recognized as consistent clinical predictors, while genetic effects require further investigation [44]. Our data indicate that MICA may interact with HLA class I to modulate NK cell functions in Taiwanese. Taken together, our data suggest that the MICA*019 may be a common major risk factor for AS susceptibility in Asians, albeit the exact mechanisms of MICA on bone remodeling in syndesmophyte formations remain to be elucidated.

Soluble MICA (sMICA) is generated by proteolytic shedding from the membrane-bound MICA of various cells. Levels of sMICA were significantly increased in rheumatoid arthritis (RA) patients, as compared to normal healthy controls, and RA patients with MICA-129Val/Val genotype had significantly higher sMICA levels than those with the MICA-129Met/Val or MICA-129Met/Met genotype [45]. More importantly, high levels of sMICA were significantly associated with severe deformed RA phenotype in south Indian Tamil population [46]. In this study, we found that AS patients produced significantly high levels of sMICA, as compared to healthy controls. In addition, AS patients with the homozygous MICA*019/019 genotype produced the highest level of sMICA among AS patients. In vitro experiments confirmed that MICA*019 cells produced the highest level of sMICA among cells expressing individual *MICA* alleles. Interestingly, MICA*008 cells produced lower levels of sMICA than MICA*019 cells. However, MICA*008 cells produced more sMICA than MICA*002 cells and MICA*010 cells, which may explain the observation that a high level of sMICA was produced in subjects carrying MICA-129Val [45], as both MICA*019 and MICA*008 contain 129Val, while MICA*002 has 129Met (Supplementary Table S2). Soluble MICA promote inflammation through the increased production of IFN-γ by activated NK cells [45]. Therefore, it is reasonable to assume that the increased production of sMICA in AS and RA patients may exacerbate inflammatory responses and perpetuate inflammation in patients. Nevertheless, this study has limitations. First, this cross-sectional study cannot assess the longitudinal effect of sMICA on AS disease progression. Second, the exact mechanism for MICA to affect bone remodeling in syndesmophyte formations remains to be elucidated.

MICA*008 is the most frequent *MICA* allele in Taiwanese (Table 1). MICA*008 cells produced significantly more sMICA than MICA*002 cells and MICA*010 cells (Figure 2D). However,MICA*008 is associated with protection against AS in Taiwanese, similarly to MICA*002 and MICA*010. MICA*008 and MICA*019 are almost identical in the extracellular domains, but very different in the C-terminal [25]. MICA*008 contains an insertion of guanine at codon 295 (*MICA* *A5.1)* that results in a truncated MICA protein lacking part of the transmembrane domain and the entire cytoplasmic tail with a premature stop codon at position 304 [47]. MICA*008 is initially synthesized as a soluble protein, which is either secreted as sMICA by exocytosis, or processed with the attachment of GPI (glycosylphosphatidylinositol) for exosomal MICA production and for membrane surface expression [25]. We confirmed that MICA*008 cells produced the highest amount of exosomal MICA among four *MICA* alleles (MICA*002, MICA*008, MICA*010 and MICA*019) (Figure 3). Exosomes containing MICA*008 downregulate NKG2D and inhibit functions of effectors cells [48,49], which may be the mechanism underlying the association of MICA*008 with the protection against AS.

Although the MICA protein could be detected in MICA*010 cell lysates in both C1R and LCL cells, MICA*010 cells did not produce detectable amounts of exosomal MICA and sMICA (Figure 2B–D) and failed to express on cell surface (Figure 2A). Our data demonstrate that the MICA*10 protein is trapped intracellularly and is unable to express functional MICA, either in soluble form or in membrane-bound MICA, confirming that MICA*010 is a non-functional MICA allele, as previously reported [30].

In summary, the MICA*019 allele is a major risk factor for AS and disease severity in Taiwanese. AS patients produce significantly high levels of sMICA, which may have a role in the pathogenesis of AS.

## Figures and Tables

**Table 1:** Comparison of MICA allele frequencies between Taiwanese normal healthy controls and AS patients.

MICA Allele	Estimated Frequency Trend Test			Logistic Regression		Logistic Regression Adjusted for Sex	
	AS(2N = 1790)	Control (2N = 1792)	*p* Value	*P_FDR_* Value	OR (95% CI)	*P_FDR_* Value	OR (95% CI)
MICA*019:01	765 (42.74%)	157 (8.76%)	<0.00001	1.91×10^−115^	14.86 (11.80–18.71)	2.25 × 10^−115^	14.90 (11.83–18.77)
MICA*008:01:01	332 (18.55%)	510 (28.46%)	2.0 × 10^−12^	1.4×10^−11^	0.57 (0.48–0.66)	8.98 × 10^−12^	0.56 (0.48–0.66)
MICA*010:01	192 (10.73%)	287 (16.02%)	1.86 × 10^−6^	4.77×10^−6^	0.62 (0.50–0.75)	4.50 × 10^−6^	0.61 (0.50–0.75)
MICA*002:01	190 (10.61%)	376 (20.98%)	3.82 × 10^−17^	8.05×10^−16^	0.45 (0.37–0.54)	7.33 × 10^−16^	0.44 (0.37–0.54)
MICA*004	73 (4.08%)	134 (7.48%)	1.70 × 10^−5^	4.06×10^−5^	0.53 (0.40–0.71)	4.98 × 10^−5^	0.54 (0.40–0.72)
MICA*012:01	55 (3.07%)	144 (8.04%)	1.49 × 10^−10^	1.92×10^−9^	0.37 (0.27–0.51)	2.11 × 10^−9^	0.37 (0.27–0.51)
MICA*045	51 (2.85%)	62 (3.46%)	0.343	0.334	0.82 (0.57–1.19)	0.360	0.83 (0.57–1.20)
MICA*033	39 (2.18%)	2 (0.11%)	1.17 × 10^−8^	6.46×10^−5^	19.36 (4.66–80.37)	6.71 × 10^−5^	19.23 (4.63–79.86)
MICA*007:01	26 (1.45%)	19 (1.06%)	0.291	0.336	1.38 (0.76–2.51)	0.360	1.38 (0.76–2.51)
MICA*018:01	5 (0.28%)	4 (0.22%)	0.738	0.738	1.25 (0.34–4.68)	0.784	1.20 (0.32–4.51)
others	62 (3.46%)	97 (5.41%)					

**Table 2:** Comparison of MICA allele frequencies between AS patients positive for syndesmophyte formation (Synd^+^) and AS patients negative for syndesmophyte formation (Synd^−^).

MICA Allele	Estimated Frequency Trend Test			Logistic Regression		Logistic Regression Adjusted for Sex	
	Synd^+^ (2N = 732)	Synd^−^ (2N = 1058)	*p* Value	P_FDR_ Value	OR (95% CI)	P_FDR_ Value	OR (95% CI)
MICA*019:01	343 (46.86%)	422 (39.89%)	8.72×10^−5^	0.001	1.68 (1.29–2.19)	0.0017	1.69 (1.29–2.22)
MICA*008:01:01	119 (16.26%)	213 (20.13%)	0.030	0.077	0.75 (0.57–0.97)	0.120	0.77 (0.59–1.01)
MICA*010:01	65 (8.88%)	127 (12.00%)	0.029	0.077	0.70 (0.50–0.97)	0.120	0.71 (0.51–1.00)
MICA*002:01	72 (9.84%)	118 (11.15%)	0.352	0.401	0.86 (0.63–1.19)	0.518	0.87 (0.63–1.21)
MICA*004	40 (5.46%)	33 (3.12%)	0.0196	0.071	1.81 (1.13–2.92)	0.081	1.84 (1.12–3.02)
MICA*012:01	26 (3.55%)	29 (2.74%)	0.397	0.401	1.32 (0.76–2.28)	0.518	1.27 (0.72–2.22)
MICA*045	14 (1.91%)	37 (3.50%)	0.063	0.105	0.54 (0.29–1.01)	0.120	0.53 (0.28–1.00)
MICA*033	16 (2.19%)	23 (2.17%)	1	0.987	1.01 (0.53–1.90)	0.841	1.07 (0.55–2.07)
MICA*007:01	7 (0.96%)	19 (1.80%)	0.199	0.247	0.52 (0.22–1.26)	0.191	0.49 (0.20–1.19)
MICA*018:01	1 (0.14%)	4 (0.38%)	0.641	0.401	0.36 (0.04–3.23)	0.540	0.45 (0.05–4.31)
others	29 (3.96%)	33 (3.12%)					

**Table 3:** Comparison of *MICA* allele frequencies between HLA-B27^+^ and HLA-B27^−^ AS patients.

MICA Allele	Estimated Frequency Trend Test			Logistic Regression		Logistic Regression Adjusted for Sex	
	B27^+^ (2N = 1568)	B27^−^ (2N = 222)	*p* Value	P_FDR_ Value	OR (95% CI)	P_FDR_ Value	OR (95% CI)
MICA*019:01	746 (47.58%)	19 (8.56%)	2.99 × 10^−50^	6.71 × 10^−34^	28.49 (16.72–48.53)	1.45 × 10^−33^	28.79 (16.83–49.26)
MICA*008:01:01	274 (17.47%)	58 (26.13%)	0.001	0.002	0.55 (0.39–0.79)	0.004	0.57 (0.40–0.81)
MICA*010:01	148 (9.44%)	44 (19.82%)	1.01 × 10^−6^	1.02 × 10^−5^	0.39 (0.26–0.58)	1.78 × 10^−5^	0.39 (0.26–0.59)
MICA*002:01	138 (8.80%)	52 (23.42%)	5.47 × 10^−12^	7.18 × 10^−10^	0.27 (0.18–0.41)	9.17 × 10^−10^	0.27 (0.18–0.41)
MICA*004	57 (3.64%)	16 (7.21%)	0.025	0.022	0.48 (0.27–0.86)	0.018	0.47 (0.26–0.84)
MICA*012:01	47 (3.00%)	8 (3.60%)	0.724	0.688	0.82 (0.38–1.79)	0.692	0.79 (0.36–1.73)
MICA*045	35 (2.23%)	16 (7.21%)	0.001	0.0003	0.29 (0.16–0.55)	0.0003	0.29 (0.16–0.54)
MICA*033	39 (2.49%)	0 (0.00%)	0.001	0.999	(0.00–Inf)	0.999	(0.00–Inf)
MICA*007:01	24 (1.53%)	2 (0.90%)	0.720	0.665	1.72 (0.40–7.38)	0.692	1.68 (0.39–7.25)
MICA*018:01	4 (0.26%)	1 (0.45%)	0.964	0.688	0.56 (0.06–5.09)	0.792	0.66 (0.07–6.09)
other	56 (3.57%)	6 (2.70%)					

## Data Availability

Not applicable.

## Supplementary Materials

The following are available online at https://www.mdpi.com/article/10.3390/jpm11060564/s1, Table S1: Demographical information of Taiwanese AS patients and normal healthy controls, Table S2: MICA non-synonymous cSNPs and major alleles identified in Taiwanese normal healthy controls and AS patients, Table S3: The LD (r2) between cSNPs and MICA alleles in Taiwanese normal healthy controls and AS patients, Table S4: Association of MICA cSNP genotypes and alleles with AS susceptibility in Taiwanese, Table S5: Association of MICA cSNP genotypes and alleles with HLA-B27 positivity in AS patients, Table S6: Comparison of MICA alleles between AS patients and normal controls with HLA-B27 positive individuals, Table S7: Comparison of MICA alleles between AS patients and normal controls with HLA-B27 negative individua. Supplementary data accompanies this paper.

## Abbreviations

AS	ankylosing spondylitis
SpA	spondyloarthritides;
mSASSS	modified Stoke Ankylosing Spondylitis Spinal Score
HLA-B27	human leukocyte antigen B27
MICA	major histocompatibility complex class I chain-related gene A
NKG2D	receptor natural killer group 2, member D
SNP	single nucleotide polymorphism
sMICA	soluble MICA
