# Supplementary material for: MICA*019 Allele and Soluble MICA as Biomarkers for Ankylosing Spondylitis in Taiwanese
Source: J Pers Med. 2021 Jun 16;11(6):564. doi: 10.3390/jpm11060564 (PMC8235541; doi:10.3390/jpm11060564)
Supplement: Supplementary file 1 [file jpm-11-00564-s001.zip › jpm-1245584-supplementary.pdf]

Online Supplementary Information

Supplemental Methods.

Supplemental Table S1. Demographical information of Taiwanese AS patients and normal healthy controls

Supplemental Table S2. *MICA* non−synonymous cSNPs and major alleles identified in Taiwanese normal healthy controls and AS patients

Supplemental Table S3. The LD (r2) between cSNPs and MICA alleles in Taiwanese normal healthy controls and AS patients

Supplemental Table S4. Association of *MICA* cSNP genotypes and alleles with AS susceptibility in Taiwanese

Supplemental Table S5. Association of *MICA* cSNP genotypes and alleles with HLA−B27 positivity in AS patients

Supplemental Table S6. Comparison of *MICA* alleles between AS patients and normal controls positive for HLA−B27

Supplemental Table S7. Comparison of *MICA* alleles between AS patients and normal controls negative for HLA−B27

Supplemental Methods.

DNA sequence analysis of *MICA* gene

Because *MICA* is highly homologous to *MICB* at nucleotide levels, we designed and validated *MICA* gen×10−specific PCR primers for the specific amplification of *MICA* gene. PCR was carried out to amplify the *MICA*−specific genomic DNA containing exon 2, 3, 4, and 5 using the sense primer (5′−CAA GAC CTT CCT TCC ACC ACC T−3′) and antisense primer (5′−CCT TGT CAC CAA CAT GCC TAT CTT T−3′). The PCR products of *MICA* DNA fragment (2352 base pairs) were treated with ExoSAP−IT (Affymetrix, Santa Clara, CA) and subsequently assessed by Sanger sequencing on an ABI 3730xl DNA Analyzer using BigDye v3.1 Sequencing kit (Applied Biosystems) and three sequencing primers to cover three *MICA* exons (sequencing primer #1: 5′−CAG CAG ACC TGT GTG TTA A−3′ for the exon 2; sequencing primer #2: 5′−GGT GAT GGG TTC GGG AA−3′ for the exon 3; and sequencing primer #3: 5′−TTC CTC TCC CCT CCT TAG A−3′ for the exon 4). IMGT/HLA database (ftp://ftp.ebi.ac.uk/pub/databases/ipd/imgt/hla/fasta/MICA_nuc.fasta, release date: 2020) are used to assign 105 *MICA* haplotypes or alleles.

Determination of soluble MICA (sMICA) levels in serum samples

Serum samples of normal healthy controls and AS patients were used in ELISA analyses of sMICA. Serum sMICA levels were determined in triplicates using a sandwich MICA DuoSet ELISA kit (R&D Systems, Minneapolis, MN, USA) according to the manufacturer’s instructions.

Generation of human MICA gene expression constructs

MICA cDNAs of peripheral blood mixed mononuclear cells from the carriers of major *MICA* alleles (*MICA*002*, *MICA*008*, *MICA*010*, and *MICA*019*) in Taiwanese were amplified by RT−PCR using the upper primer 5′−CA GCA **GAA TTC** GTC GGG GCC ATG GGG CTG GGC CCG GTC−3′ and the lower primer 5′−CAG **GGA TCC** CTA GGC GCC CTC AGT GGA G−3′ (for *MICA*008* allele, the lower primer 5′−CAG **GGA TCC** CTA GGT GCC CTC AGT GGA G−3′ were used instead). *MICA* cDNAs were subsequently cloned into the lentiviral vector pCDH−CMV−EF1−copGFP (Systems Biosciences). Pseudo−lentiviral particles were produced and subsequently used to transduce target cells according to the vendor’s instructions (System Biosciences)

Generation of cell lines expressing *MICA* alleles

The C1R cells (ATCC#CRL−1573, Manassas, VA) and LCL−721.221 cells (ATCC#CRL−1855) were maintained in the DMEM medium supplemented with 10% fetal calf serum and 1% GlutaMax (Invitrogen) in 5% CO_2. Transduced cells were sorted on vFACSAria III cytometer (BD Biosciences, Mountain View, CA) for copGFP−positive cell population. Established stable C1R cell lines expressing empty vector, *MICA*002*, *MICA*008*, *MICA*010*, or *MICA*019* alleles were continuously cultured and monitored for GFP expressions before analyses of sMICA, exosomal MICA, and cellular MICA.

Flow Cytometry analyses of membran×10−bound MICA

To detect surface MICA, the parental C1R and LCL−721.221 cells along with their respective stable clones expressing empty vector, *MICA*002*, *MICA*008*, *MICA*010*, and *MICA*019* were stained with APC−conjugated anti−human MICA/MICB (Biolegend, San Diego, CA, USA) for 30 min at 4°C and analyzed on a EC800 (SONY) system. The transduced GFP^+ cells were gated for MICA expression analyses while the parental cells (GFP−) were used as the negative control for transduction.

Western analyses and detection of sMICA, exosomal MICA, and cellular MICA

All cells were cultured in RPMI containing 1% FBS for 24−hrs before the harvest of cells and cell−free culture supernatants. To isolate exosomes, 24−hr cell−free culture supernatants were subjected to centrifugation at 10,000 ×g for 1 hr and the resultant supernatants were further subjected to ultracentrifugation at 100,000 ×g for 1 hr. The ultracentrifugation pellets containing exosomes were subsequently used for Western blot analyses of exosomal MICA. The ultracentrifugation supernatant fractions containing sMICA were immunoprecipitated with anti−human MICA/MICA (Biolegend) and protein G Mag sepharose Xtra beads (GE Healthcare) to concentrate sMICA for Western blot analysis. Total cell lysates were solubilized in lysis buffer (1% Triton X−100, 50 mM Tris−Cl, pH 7.4, 300 mM NaCl, 5 mM EDTA, 0.02% NaN_3) containing complete protease inhibitors (Roche Applied Science) for 30 min on ice. The immunoprecipitation−concentrated sMICA, the isolated exosomes, and total cell lysate were subjected to gel−electrophoresis on 4–12% gradient SDS−PAGE under reducing conditions and transferred to PVDF membranes (GE Healthcare). The membrane was blocked by 5% BSA in PBST and MICA proteins were detected by incubating the membrane with rabbit anti−human MICA antibody (Abcam) at 4 °C overnight, followed by incubation with alkaline phosphatas×10−conjugated goat anti−rabbit secondary antibodies at room temperature for 1 hr. MICA proteins were visualized using ECF™ substrates (GE Healthcare) and then detected by imaging system Typhoon FLA 9500 (GE Healthcare). The blots were further r×10−probed with the primary antibodies against exosome marker CD55 (Santa Cruz Biotechnology), ER marker calnexin (Cell Signaling Technology) or cytoplasmic protein GAPDH (Epitomics) followed by their respective alkaline phosphatas×10−conjugated secondary antibodies.

Statistical analysis

We carried out singl×10−locus analyses of *MICA* cSNPs to compare distributions of genotypes and alleles between normal healthy controls and AS patients. Three chi−square tests: the genotype test, the allele test, and the Cochran−Armitage trend test were performed, and associations with cSNPs were identified using the SAS/Genetics software package release 8.2 (SAS Institute, Cary, NC). Based on the identified risk allele, p−values, odds ratios (ORs), and 95% confidence interval (CIs) were then calculated. To account for the multiple testing corrections, the FDR−corrected p−values were generated by using False Discovery Rate (FDR) correction that accomplished by the modified version of FDR programmed in QVALUE software (http://genomics.princeton.edu/storeylab/qvalue/ (accessed on 2020 October 1st)). Linkage disequilibrium (LD) between marker loci or cSNPs was measured and haplotype blocks were constructed using Haploview 4.2 (Broad Institute, Cambridge, MA, USA; http://www.broad.mit.edu/mpg/haploview (accessed on 2020 October 1st)). Association of the estimated haplotypes or alleles and disease status

were tested in logistic regression models. To investigate the genetic association with clinical characteristics, we controlled for each of clinical characteristics and performed stepwise logistic regression analyses. Unpaired t–tests of GraphPad Prism 6.0 (GraphPad, La Jolla, CA, USA) were used to compare serum sMICA levels between normal healthy controls and AS patients and between groups of subjects with different genotypes containing at least one *MICA*019* allele. The 5% level of significance for p–values was used for all the analyses

Table S1. Demographical information of Taiwanese AS patients and normal healthy controls .

Characteristics	AS Case *N = 895*	Normal N = 896	Total N = 1791
Gender, N (%)			
Male	725/895 (81.0%)	752/896 (83.9%)	1477/1791 (82.46%)
Female	170/895 (19.0%)	144/896 (16.1%)	314/1791 (17.53%)
Age onset, Mean ± Std.	25.34 ± 9.99		
Bamboo (Syndesmophyte)			
no syndesmophytes	529/895 (59.1%)		
mSASSS < 24	114/895 (12.7%)		
mSASSS ≥ 24	252/895 (28.2%)		
B27 final (HLA–B27)			
Positive	774/895 (86.5%)	41/879 (4.7%)	
Negative	121/895 (13.5%)	838/879 (95.3%)	

Table S2. *MICA* non–synonymous cSNPs and major alleles identified in Taiwanese normal healthy controls and AS patients.

Residue Position	SNP ID Number	Codon Changes	Exon	MICA Allele									
				001 *	002	008	010	019	045	007	004	033	012
6R > P	rs9380254	C*G*T/C*C*T	**Exon 2**	**G**	–	–	C	–	–	–	–	–	–
14W > G	rs1063630	*T*GG/*G*GG	**Exon 2**	**T**	G	–	–	–	–	–	–	–	–
24A > T	rs1051785	*G*CT/*A*CT	**Exon 2**	**A**	G	G	G	G	G	G	G	G	–
36Y > C	rs1051786	T*A*T/T*G*T	**Exon 2**	**G**	–	A	A	A	–	–	A	A	–
122L > V	rs1051790	*C*TG/*G*TG	**Exon 3**	**C**	–	–	–	–	–	–	G	–	–
124T > S	rs41539919	*A*CT/*T*CT	**Exon 3**	**A**	–	–	–	–	–	–	–	T	–
** 125E > K	**rs1051791**	*G*AG/*A*AG	**Exon 3**	**A**	G	G	G	G	G	G	G	G	G
129V > M	rs1051792	*G*TG/*A*TG	**Exon 3**	**A**	–	G	G	G	–	–	G	G	–
156H > L	rs3819268	C*A*C/C*T*C	**Exon 3**	**A**	–	–	–	–	–	–	–	–	T
173E > K	rs1051794	*G*AA/*A*AA	**Exon 3**	**A**	–	G	G	G	–	–	G	G	–
175G > S	rs1131896	*G*GC/*A*GC	**Exon 3**	**G**	–	–	A	A	–	–	A	A	–
181T > R	rs1131897	A*C*A/A*G*A	**Exon 3**	**C**	–	–	–	–	–	–	G	–	–
**206S > G**	rs1131898	*A*GC/*G*GC	**Exon 4**	**G**	–	A	A	A	A	–	A	A	–
**210R > W**	rs1051798	*C*GG/*T*GG	**Exon 4**	**T**	–	C	C	C	C	–	C	C	–
213I > T	rs1140700	A*T*A/A*C*A	**Exon 4**	**C**	–	T	T	T	T	–	–	–	–
**215S > T**	rs1051799	A*C*C/A*G*C	**Exon 4**	**G**	–	C	C	C	C	–	C	C	–
251Q > R	rs1063635	C*A*A/C*G*A	**Exon 4**	**A**	–	G	G	G	–	–	–	G	–

* MICA*001 allele was listed as the reference allele for the nucleotide sequence comparison with other MICA alleles that had allele frequency >1% in the combined population of Taiwanese normal controls and AS patients as shown in the Table 1. ** rs1051791 that causes amino acid (aa) substation at the residue 125 was not detected in Taiwanese but is listed to compare the nucleotide differences between MICA*001 and major MICA alleles identified in Taiwanese. Non–conservative residue changes are underlined. Residue substitutions that define two MICA lineages are in bold.

Table S3. The LD (r2) between cSNPs and MICA alleles in Taiwanese normal healthy controls and AS patients.

SNP ID Number	Exon	MICA Allele								
		002	008	010	019	045	007	004	033	012
rs9380254	**Exon 2**	0.031	0.051	0.934	0.057	0.005	0.001	0.010	0.002	0.010
rs1063630	**Exon 2**	0.952	0.061	0.030	0.068	0.006	0.003	0.012	0.001	0.012
rs1063631	**Exon 2**	0.481	0.120	0.060	0.135	0.083	0.033	0.024	0.004	0.151
rs1051785	**Exon 2**	0.012	0.020	0.010	0.022	0.001	0.001	0.003	0.001	0.908
rs17200158	**Exon 2**	0.000	0.001	0.000	0.001	0.000	0.000	0.000	0.000	0.000
rs1051786	**Exon 2**	0.481	0.120	0.060	0.135	0.084	0.033	0.024	0.004	0.151
rs1063632	**Exon 2**	0.012	0.020	0.010	0.023	0.001	0.001	0.004	0.001	0.899
rs41557113	**Exon 3**	0.000	0.001	0.001	0.001	0.000	0.001	0.000	0.000	0.000
rs41556715	**Exon 3**	0.000	0.001	0.000	0.001	0.000	0.000	0.000	0.000	0.000
rs1051790	**Exon 3**	0.012	0.020	0.010	0.022	0.002	0.001	0.951	0.001	0.004
rs41539919	**Exon 3**	0.001	0.004	0.002	0.004	0.000	0.001	0.001	0.976	0.001
rs1051792	**Exon 3**	0.482	0.120	0.060	0.135	0.084	0.033	0.024	0.004	0.151
rs3819268	**Exon 3**	0.012	0.020	0.010	0.022	0.002	0.001	0.004	0.001	0.926
rs1051794	**Exon 3**	0.468	0.123	0.062	0.139	0.081	0.032	0.025	0.003	0.147
rs1131896	**Exon 3**	0.168	0.275	0.172	0.387	0.029	0.011	0.068	0.013	0.053
rs17206680	**Exon 4**	0.011	0.018	0.009	0.021	0.548	0.214	0.004	0.000	0.003
rs1051796	**Exon 4**	0.480	0.120	0.060	0.136	0.083	0.033	0.024	0.004	0.150
rs1051797	**Exon 4**	0.485	0.119	0.060	0.134	0.084	0.033	0.024	0.003	0.152
rs1140700	**Exon 4**	0.375	0.154	0.077	0.174	0.065	0.025	0.123	0.006	0.117
rs1051799	**Exon 4**	0.491	0.118	0.059	0.133	0.085	0.033	0.023	0.003	0.154
rs41554616	**Exon 4**	0.007	0.011	0.005	0.012	0.924	0.000	0.002	0.000	0.001
rs1063635	**Exon 4**	0.364	0.159	0.080	0.179	0.063	0.025	0.119	0.006	0.114

Table S4. Association of *MICA* cSNP genotypes and alleles with AS susceptibility in Taiwanese.

SNP	Risk Allele Frequency	Genotype Frequency			P_{Trend} *	P_{FDR}	Test for Mode of Inheritance Unadjusted				Test For Mode Of Inheritance Adjusted For Sex		
								p	P_{FDR}	OR (95% CI)	p	P_{FDR}	OR (95% CI)
rs9380254 (G/C)	G	GG	GC	CC			Additive	1.47×10^{-6}	2.15×10^{-6}	1.62 (1.33–1.97)	1.47×10^{-6}	2.15×10^{-6}	1.62 (1.33–1.97)
case	1586 (88.60%)	699 (78.10%)	188 (21.01%)	8 (0.89%)	<0.0001	<0.0001	GG + GC vs. CC	0.0193	0.0305		2.66 (1.17–6.04) 0.0186	0.0341	2.68 (1.18–6.08)
control	1488 (83.04%)	613 (68.42%)	262 (29.24%)	21 (2.34%)			GG vs. GC + CC	4.02×10^{-6}	5.90×10^{-6}	1.65 (1.33–2.04)	4.10×10^{-6}	6.01×10^{-6}	1.65 (1.33–2.03)
rs1063630 (T/G)	T	TT	TG	GG			Additive	2.36×10^{-17}	4.73×10^{-17}	2.27 (1.88–2.75)	2.22×10^{-17}	4.44×10^{-17}	2.27 (1.88–2.75)
case	1592 (88.94%)	702 (78.44%)	188 (21.01%)	5 (0.56%)	<0.0001	<0.0001	TT + TG vs. GG	1.70×10^{-6}	2.94×10^{-6}	9.63 (3.81–24.36)	1.68×10^{-6}	3.36×10^{-6}	9.65 (3.82–24.41)
control	1400 (78.13%)	550 (61.38%)	300 (33.48%)	46 (5.13%)			TT vs. TG + GG	7.14×10^{-15}	1.43×10^{-14}	2.29 (1.86–2.82)	6.73×10^{-15}	1.35×10^{-14}	2.29 (1.86–2.82)
rs1063631 (T/C)	T	TT	TC	CC			Additive	1.05×10^{-26}	2.56×10^{-26}	2.39 (2.04–2.81)	1.27×10^{-26}	3.10×10^{-26}	2.39 (2.04–2.80)
case	1434 (80.11%)	558 (62.35%)	318 (35.53%)	19 (2.12%)	<0.0001	<0.0001	TT + TC vs. CC	1.68×10^{-14}	3.59×10^{-14}	6.92 (4.22–11.35)	1.80×10^{-14}	4.41×10^{-14}	6.91 (4.22–11.33)

control	1142 (63.73%)	363 (40.51%)	416 (46.43%)	117 (13.06%)			TT vs. TC + CC	4.83×10^{-20}	1.52×10^{-19}	2.43 (2.01–2.94)	5.69×10^{-20}	1.79×10^{-19}	2.43 (2.01–2.94)
rs1051785 (G/A)	G	GG	GA	AA			Additive	1.91×10^{-9}	3.23×10^{-9}	2.51 (1.86–3.39)	1.99×10^{-9}	3.36×10^{-9}	2.51 (1.86–3.39)
case	1726 (96.42%)	832 (92.96%)	62 (6.93%)	1 (0.11%)	<0.0001	<0.0001	GG + GA vs. AA	0.0494	0.0587	8.05 (1.01–64.53)	0.0504	0.0693	7.99 (1.00–64.00)
control	1638 (91.41%)	750 (83.71%)	138 (15.40%)	8 (0.89%)			GG vs. GA + AA	2.83×10^{-9}	4.78×10^{-9}	2.57 (1.88–3.51)	2.91×10^{-9}	4.93×10^{-9}	2.57 (1.88–3.51)
rs17200158 (T/G)	T	TT	TG	GG			Additive	0.4234	0.4234	2.00 (0.37–10.96)	0.4581	0.4581	1.90 (0.35–10.45)
case	1788 (99.89%)	893 (99.78%)	2 (0.22%)	0 (0.00%)	0.676	0.676	TT + TG vs. GG	NaN	NaN	1.00 (1.00–1.00)	0.2367	0.2604	1.20 (0.89–1.61)
control	1788 (99.78%)	892 (99.55%)	4 (0.45%)	0 (0.00%)			TT vs. TG + GG	0.4234	0.4436	2.00 (0.37–10.96)	0.4581	0.4786	1.90 (0.35–10.45)
rs1051786 (A/G)	A	AA	AG	GG			Additive	7.29×10^{-27}	2.55×10^{-26}	2.40 (2.04–2.81)	8.79×10^{-27}	2.97×10^{-26}	2.40 (2.04–2.81)
case	1435 (80.17%)	559 (62.46%)	317 (35.42%)	19 (2.12%)	<0.0001	<0.0001	AA + AG vs. GG	1.68×10^{-14}	3.59×10^{-14}	6.92 (4.22–11.35)	1.80×10^{-14}	4.41×10^{-14}	6.91 (4.22–11.33)
control	1142 (63.73%)	363 (40.51%)	416 (46.43%)	117 (13.06%)			AA vs. AG + GG	3.14×10^{-20}	1.15×10^{-19}	2.44 (2.02–2.95)	3.68×10^{-20}	1.35×10^{-19}	2.44 (2.02–2.95)
rs1063632 (G/A)	G	GG	GA	AA			Additive	4.54×10^{-9}	7.14×10^{-9}	2.43 (1.81–3.28)	4.77×10^{-9}	7.50×10^{-9}	2.43 (1.81–3.28)
case	1724 (96.31%)	830 (92.74%)	64 (7.15%)	1 (0.11%)	<0.0001	<0.0001	GG + GA vs. AA	0.0494	0.0587	8.05 (1.01–64.53)	0.0504	0.0693	7.99 (1.00–64.00)
control	1638 (91.41%)	750 (83.71%)	138 (15.40%)	8 (0.89%)			GG vs. GA + AA	7.23×10^{-9}	1.14×10^{-8}	2.49 (1.83–3.38)	7.52×10^{-9}	1.18×10^{-8}	2.48 (1.82–3.38)
rs41557113 (G/A)	G	GG	GA	AA			Additive	0.0166	0.0203	12.14 (1.57–93.53)	0.0177	0.0216	11.85 (1.54–91.34)
case	1789 (99.94%)	894 (99.89%)	1 (0.11%)	0 (0.00%)	0.0024	0.0029	GG + GA vs. AA	NaN	NaN	1.00 (1.00–1.00)	0.2367	0.2604	1.20 (0.89–1.61)
control	1780 (99.33%)	884 (98.66%)	12 (1.34%)	0 (0.00%)			GG vs. GA + AA	0.0166	0.0203	12.14 (1.57–93.53)	0.0177	0.0216	11.85 (1.54–91.34)
rs41556715 (G/A)	G	GG	GA	AA			Additive	0.2730	0.3003	2.51 (0.48–12.95)	0.2869	0.3156	2.44 (0.47–12.62)
case	1788 (99.89%)	893 (99.78%)	2 (0.22%)	0 (0.00%)	0.4547	0.4890	GG + GA vs. AA	NaN	NaN	1.00 (1.00–1.00)	0.2367	0.2604	1.20 (0.89–1.61)
control	1787 (99.72%)	891 (99.44%)	5 (0.56%)	0 (0.00%)			GG vs. GA + AA	0.2730	0.3003	2.51 (0.48–12.95)	0.2869	0.3156	2.44 (0.47–12.62)
rs1051790 (C/G)	C	CC	CG	GG			Additive	2.05×10^{-5}	2.82×10^{-5}	1.86 (1.40–2.47)	2.54×10^{-5}	3.49×10^{-5}	1.84 (1.39–2.45)
case	1713 (95.70%)	819 (91.51%)	75 (8.38%)	1 (0.11%)	<0.0001	<0.0001	CC + CG vs. GG	0.0366	0.0535	9.07 (1.15–71.75)	0.0362	0.0613	9.12 (1.15–72.18)

control	1652 (92.19%)	765 (85.38%)	122 (13.62%)	9 (1.00%)			CC vs. CG + GG	6.02×10^{-5}	7.79×10^{-5}	1.85 (1.37–2.49)	7.55×10^{-5}	9.77×10^{-5}	1.83 (1.36–2.47)
rs41539919 (A/T)	T	AA	AT	TT			Additive	3.79×10^{-5}	4.91×10^{-5}	19.90 (4.80–82.56)	3.93×10^{-5}	5.08×10^{-5}	19.79 (4.77–82.09)
case	40 (2.23%)	856 (95.64%)	38 (4.25%)	1 (0.11%)	<0.0001	<0.0001	TT + ATvs. AA	3.35×10^{-5}	4.61×10^{-5}	20.37 (4.90–84.60)	3.46×10^{-5}	4.76×10^{-5}	20.26 (4.88–84.19)
control	2 (0.11%)	894 (99.78%)	2 (0.22%)	0 (0.00%)			TT vs. AT + AA	1.0000	1.0000	Inf (0.00–Inf)	1.0000	1.0000	Inf (0.00–Inf)
rs1051792 (G/A)	G	GG	GA	AA			Additive	2.39×10^{-27}	1.05×10^{-26}	2.42 (2.06–2.84)	2.88×10^{-27}	1.27×10^{-26}	2.42 (2.06–2.84)
case	1437 (80.28%)	561 (62.68%)	315 (35.20%)	19 (2.12%)	<0.0001	<0.0001	GG + GA vs. AA	1.68×10^{-14}	3.59×10^{-14}	6.92 (4.22–11.35)	1.80×10^{-14}	4.41×10^{-14}	6.91 (4.22–11.33)
control	1141 (63.67%)	362 (40.40%)	417 (46.54%)	117 (13.06%)			GG vs. GA + AA	8.55×10^{-21}	3.76×10^{-20}	2.48 (2.05–3.00)	1.00×10^{-20}	4.40×10^{-20}	2.47 (2.05–2.99)
rs3819268 (A/T)	A	AA	AT	TT			Additive	7.04×10^{-10}	1.29×10^{-9}	2.62 (1.93–3.55)	8.04×10^{-10}	1.47×10^{-9}	2.61 (1.92–3.54)
case	1729 (96.59%)	835 (93.30%)	59 (6.59%)	1 (0.11%)	<0.0001	<0.0001	AA + AT vs. TT	0.0494	0.0587	8.05 (1.01–64.53)	0.0504	0.0693	7.99 (1.00–64.00)
control	1639 (91.46%)	751 (83.82%)	137 (15.29%)	8 (0.89%)			AA vs. AT + TT	9.41×10^{-10}	1.73×10^{-9}	2.69 (1.96–3.69)	1.07×10^{-9}	1.97×10^{-9}	2.68 (1.95–3.68)
rs1051794 (G/A)	G	GG	GA	AA			Additive	1.91×10^{-28}	1.05×10^{-27}	2.46 (2.10–2.88)	2.39×10^{-28}	1.32×10^{-27}	2.45 (2.09–2.88)
case	1431 (79.94%)	557 (62.23%)	317 (35.42%)	21 (2.35%)	<0.0001	<0.0001	GG + GA vs. AA	6.52×10^{-15}	2.48×10^{-14}	6.56 (4.09–10.53)	7.28×10^{-15}	3.20×10^{-14}	6.54 (4.07–10.50)
control	1126 (62.83%)	352 (39.29%)	422 (47.10%)	122 (13.62%)			GG vs. GA + AA	6.26×10^{-22}	3.44×10^{-21}	2.55 (2.11–3.08)	7.43×10^{-22}	4.09×10^{-21}	2.54 (2.10–3.08)
rs1131896 (G/A)	A	GG	GA	AA			Additive	1.96×10^{-56}	4.31×10^{-55}	3.90 (3.30–4.62)	1.23×10^{-56}	2.71×10^{-55}	3.92 (3.31–4.64)
case	1092 (61.01%)	73 (8.16%)	552 (61.68%)	270 (30.17%)	<0.0001	<0.0001	AA + GA vs. GG	4.82×10^{-55}	1.06×10^{-53}	8.84 (6.72–11.62)	3.00×10^{-55}	6.59×10^{-54}	8.92 (6.78–11.73)
control	601 (33.54%)	394 (43.97%)	403 (44.98%)	99 (11.05%)			AA vs. GA + GG	4.60×10^{-22}	8.75×10^{-21}	3.48 (2.70–4.48)	3.87×10^{-22}	8.50×10^{-21}	3.49 (2.71–4.49)
rs17206680 (C/T)	C	CC	CT	TT			Additive	0.422963	0.423435	1.13 (0.84–1.50)	0.445551	0.458134	1.12 (0.84–1.49)
case	1695 (94.69%)	801 (89.50%)	93 (10.39%)	1 (0.11%)	0.4668	0.4890	CC + CT vs. TT	0.571713	0.603475	2.00 (0.18–22.10)	0.586441	0.614367	1.95 (0.18–21.53)
control	1686 (94.08%)	792 (88.39%)	102 (11.38%)	2 (0.22%)			CC vs. CT + TT	0.456311	0.456311	1.12 (0.83–1.50)	0.478649	0.478649	1.11 (0.83–1.50)
rs1051796 (C/T)	C	CC	CT	TT			Additive	8.68×10^{-26}	1.91×10^{-25}	2.35 (2.01–2.76)	1.03×10^{-25}	2.27×10^{-25}	2.35 (2.00–2.76)
case	1430 (79.89%)	553 (61.79%)	324 (36.20%)	18 (2.01%)	<0.0001	<0.0001	CC + CT vs. TT	2.32×10^{-14}	4.41×10^{-14}	7.17 (4.32–11.90)	2.44×10^{-14}	5.38×10^{-14}	7.16 (4.32–11.89)

control	1145 (63.90%)	364 (40.63%)	417 (46.54%)	115 (12.83%)			CC vs. CT + TT	6.12×10^{-19}	1.35×10^{-18}	2.36 (1.96–2.86)	7.13×10^{-19}	1.57×10^{-18}	2.36 (1.95–2.85)
rs1051797 (C/T)	C	CC	CT	TT			Additive	8.68×10^{-27}	2.55×10^{-26}	2.41 (2.05–2.82)	1.02×10^{-26}	2.97×10^{-26}	2.40 (2.05–2.82)
case	1437 (80.28%)	559 (62.46%)	319 (35.64%)	17 (1.90%)	<0.0001	<0.0001	CC + CT vs. TT	1.70×10^{-14}	3.59×10^{-14}	7.60 (4.53–12.77)	1.78×10^{-14}	4.41×10^{-14}	7.60 (4.52–12.76)
control	1146 (63.95%)	365 (40.74%)	416 (46.43%)	115 (12.83%)			CC vs. CT + TT	7.34×10^{-20}	1.79×10^{-19}	2.42 (2.00–2.93)	8.52×10^{-20}	2.08×10^{-19}	2.42 (2.00–2.92)
rs1140700 (T/C)	T	TT	TC	CC			Additive	1.06×10^{-32}	7.80×10^{-32}	2.53 (2.17–2.95)	1.52×10^{-32}	1.12×10^{-31}	2.53 (2.17–2.95)
case	1367 (76.37%)	502 (56.09%)	363 (40.56%)	30 (3.35%)	<0.0001	<0.0001	TT + TC vs. CC	4.31×10^{-21}	4.10×10^{-20}	6.85 (4.59–10.22)	5.00×10^{-21}	5.50×10^{-20}	6.83 (4.58–10.19)
control	1020 (56.92%)	296 (33.04%)	428 (47.77%)	172 (19.20%)			TT vs. TC + CC	2.50×10^{-22}	1.83×10^{-21}	2.59 (2.14–3.14)	3.44×10^{-22}	2.52×10^{-21}	2.58 (2.13–3.13)
rs1051799 (C/G)	C	CC	CG	GG			Additive	9.28×10^{-27}	2.55×10^{-26}	2.37 (2.02–2.77)	1.08×10^{-26}	2.97×10^{-26}	2.36 (2.02–2.77)
case	1444 (80.67%)	570 (63.69%)	304 (33.97%)	21 (2.35%)	<0.0001	<0.0001	CC + CG vs. GG	2.31×10^{-15}	1.10×10^{-14}	6.75 (4.21–10.82)	2.37×10^{-15}	1.30×10^{-14}	6.75 (4.21–10.82)
control	1147 (64.01%)	376 (41.96%)	395 (44.08%)	125 (13.95%)			CC vs. CG + GG	6.72×10^{-20}	1.79×10^{-19}	2.43 (2.01–2.93)	7.84×10^{-20}	2.08×10^{-19}	2.42 (2.00–2.93)
rs41554616 (C/G)	C	CC	CG	GG			Additive	0.1534	0.1776	1.30 (0.91–1.85)	0.1675	0.1940	1.29 (0.90–1.84)
case	1737 (97.04%)	843 (94.19%)	51 (5.70%)	1 (0.11%)	0.1763	0.2041	CC + CG vs. GG	0.2147	0.2399	4.01 (0.45–35.94)	0.2234	0.2604	3.91 (0.44–35.04)
control	1723 (96.15%)	831 (92.75%)	61 (6.81%)	4 (0.45%)			CC vs. CG + GG	0.2170	0.2513	1.27 (0.87–1.85)	0.2342	0.2711	1.26 (0.86–1.83)
rs1063635 (G/A)	G	GG	GA	AA			Additive	2.44×10^{-33}	2.69×10^{-32}	2.57 (2.21–3.00)	3.58×10^{-33}	3.94×10^{-32}	2.57 (2.20–3.00)
case	1356 (75.75%)	492 (54.97%)	372 (41.56%)	31 (3.46%)	<0.0001	<0.0001	GG + GA vs. AA	9.58×10^{-21}	6.06×10^{-20}	6.57 (4.43–9.76)	1.11×10^{-20}	8.16×10^{-20}	6.56 (4.42–9.73)
control	1007 (56.19%)	282 (31.47%)	443 (49.44%)	171 (19.08%)			GG vs. GA + AA	3.02×10^{-23}	3.32×10^{-22}	2.66 (2.19–3.22)	4.30×10^{-23}	4.73×10^{-22}	2.65 (2.19–3.21)

* P_{Trend} indicates the *p*–value from the Cochran–Armitage trend test with 10,000 permutations. Specifically, the cSNP rs1051792A>G was significantly associated with AS susceptibility in genotype test (AG + GG vs. AA: P_{adj} = 1.80 × 10^{-14}; P_{FDR} = 4.41 × 10^{-14}, OR 6.91, 95% CI 4.22–11.33. GG vs. AA +AG: P_{adj} = 1.0 × 10^{-20}, P_{FDR} = 4.4 × 10^{-20}, OR 2.47, 95% CI 2.05–2.99. Additive model: P_i = 2.88×10^{-27}, P_{FDR} = 1.27 × 10^{-26}, OR 2.42, 95% CI 2.06–2.84) and in allele test (trend test $p < 0.0001$). *MICA* cSNP rs1063630T>G, rs1063631T>G, rs1051786A>G, rs1051794G>A, rs1131896G>A, rs1051796C>T, rs1051797C>T, rs1140700T>C, rs1051799C>T, rs1063635G>A were also significantly associated with AS susceptibility.

Table S5. Association of *MICA* cSNP genotypes and alleles with HLA–B27 positivity in AS patients.

SNP	Risk Allele Frequency	Genotype Frequency			P_{Trend}*	P_{FDR}	Test for mode of inheritance unadjusted				Test for mode of inheritance adjusted for sex		
								P	P_{FDR}	OR (95% CI)	P	P_{FDR}	OR (95% CI)
rs9380254 (G/C)	G	GG	GC	CC			Additive	1.09×10^{-5}	1.60×10^{-5}	2.38 (1.62–3.50)	1.39×10^{-5}	2.35×10^{-5}	2.37 (1.61–3.49)
B27 positive	1409 (89.86%)	628 (80.10%)	153 (19.52%)	3 (0.38%)	<0.0001	<0.0001	GG + GC vs. CC	0.0007	0.0009	12.28 (2.89–52.13)	0.0008	0.0008	12.27 (2.85–52.84)
B27 negative	177 (79.73%)	71 (63.96%)	35 (31.53%)	5 (4.50%)			GG vs. GC + CC	0.0002	0.0002	2.27 (1.48–3.47)	0.0002	0.0003	2.26 (1.47–3.47)
rs1063630 (T/G)	T	TT	TG	GG			Additive	6.06×10^{-11}	9.52×10^{-11}	3.72 (2.51–5.51)	7.16×10^{-11}	1.31×10^{-10}	3.74 (2.52–5.56)
B27 positive	1424 (90.82%)	642 (81.89%)	140 (17.86%)	2 (0.26%)	<0.0001	<0.0001	TT + TG vs. GG	0.0094	0.0112	10.86 (1.79–65.74)	0.0091	0.0091	11.21 (1.82–68.96)
B27 negative	168 (75.68%)	60 (54.05%)	48 (43.24%)	3 (2.70%)			TT vs. TG + GG	2.08×10^{-10}	9.16×10^{-10}	3.84 (2.54–5.82)	2.54×10^{-10}	1.86×10^{-9}	3.86 (2.54–5.87)
rs1063631 (T/C)	T	TT	TC	CC			Additive	1.20×10^{-12}	2.93×10^{-12}	3.72 (2.59–5.34)	8.26×10^{-13}	2.60×10^{-12}	3.81 (2.64–5.50)
B27 positive	1296 (82.65%)	518 (66.07%)	260 (33.16%)	6 (0.77%)	<0.0001	<0.0001	TT + TC vs. CC	1.77×10^{-8}	3.74×10^{-8}	17.20 (6.39–46.29)	1.86×10^{-8}	2.73×10^{-8}	17.92 (6.56–48.99)
B27 negative	138 (62.16%)	40 (36.04%)	58 (52.25%)	13 (11.71%)			TT vs. TC + CC	4.59×10^{-9}	9.18×10^{-9}	3.46 (2.28–5.23)	2.75×10^{-9}	6.71×10^{-9}	3.56 (2.34–5.41)
rs1051785 (G/A)	G	GG	GA	AA			Additive	0.2390	0.2767	1.49 (0.77–2.91)	0.2065	0.2673	1.54 (0.79–3.03)
B27 positive	1515 (96.62%)	731 (93.24%)	53 (6.76%)	0 (0.00%)	0.2484	0.3215	GG + GA vs. AA	1.0000	1.0000	Inf (0.00– Inf)	0	0	Inf (Inf– Inf)
B27 negative	211 (95.05%)	101 (90.99%)	9 (8.11%)	1 (0.90%)			GG vs. GA + AA	0.3877	0.4490	1.37 (0.67–2.77)	0.3482	0.4506	1.41 (0.69–2.86)
rs17200158 (T/G)	T	TT	TG	GG			Additive	0	0	0.00 (0.00–0.00)	1.0000	1.0000	0.00 (0.00– Inf)
B27 positive	1566 (99.87%)	782 (99.74%)	2 (0.26%)	0 (0.00%)	1	1	TT + TG vs. GG	NaN	NaN	1.00 (1.00–1.00)	2.93×10^{-20}	6.45×10^{-20}	0.06 (0.03–0.11)
B27 negative	222 (100.00%)	111 (100.00%)	0 (0.00%)	0 (0.00%)			TT vs. TG + GG	0	0	0.00 (0.00–0.00)	1.0000	1.0000	0.00 (0.00– Inf)
rs1051786 (A/G)	A	AA	AG	GG			Additive	1.03×10^{-12}	2.82×10^{-12}	3.73 (2.60–5.36)	7.21×10^{-13}	2.60×10^{-12}	3.83 (2.65–5.52)
B27 positive	1297 (82.72%)	519 (66.20%)	259 (33.04%)	6 (0.77%)	<0.0001	<0.0001	AA + AG vs. GG	1.77×10^{-8}	3.74×10^{-8}	17.20 (6.39–46.29)	1.86×10^{-8}	2.73×10^{-8}	17.92 (6.56–48.99)
B27 negative	138 (62.16%)	40 (36.04%)	58 (52.25%)	13 (11.71%)			AA vs. AG + GG	3.92×10^{-9}	8.62×10^{-9}	3.48 (2.30–5.26)	2.39×10^{-9}	6.71×10^{-9}	3.58 (2.35–5.44)
rs1063632 (G/A)	G	GG	GA	AA			Additive	0.2852	0.3137	1.44 (0.74–2.80)	0.2436	0.2977	1.49 (0.76–2.92)
B27 positive	1513 (96.49%)	729 (92.98%)	55 (7.02%)	0 (0.00%)	0.3447	0.4213	GG + GA vs. AA	1.0000	1.0000	Inf (0.00–Inf)	0	0	Inf (Inf– Inf)
B27 negative	211 (95.05%)	101 (90.99%)	9 (8.11%)	1 (0.90%)			GG vs. GA + AA	0.4500	0.4950	1.31 (0.65–2.66)	0.3999	0.4888	1.36 (0.67–2.76)
rs41557113 (G/A)	G	GG	GA	AA			Additive	0	0	0.00 (0.00–0.00)	0.9998	1.0000	0.00 (0.00– Inf)
B27 positive	1567 (99.94%)	783 (99.87%)	1 (0.13%)	0 (0.00%)	1	1	GG + GA vs. AA	NaN	NaN	1.00 (1.00–1.00)	2.93×10^{-20}	6.45×10^{-20}	0.06 (0.03–0.11)

B27 negative	222 (100.00%)	111 (100.00 %)	0 (0.00%)	0 (0.00%)			GG vs. GA + AA	0	0	0.00 (0.00–0.00)	0.9998	1.0000	0.00 (0.00– Inf)
rs41556715 (G/A)	G	GG	GA	AA			Additive	4.09×10^{-92}	3.00×10^{-91}	0.00 (0.00–0.00)	0	0	0.00 (0.00–0.00)
B27 positive	1566 (99.87%)	782 (99.74%)	2 (0.26%)	0 (0.00%)	1	1	GG + GA vs. AA	NaN	NaN	1.00 (1.00–1.00)	2.93×10^{-20}	6.45×10^{-20}	0.06 (0.03–0.11)
B27 negative	222 (100.00%)	111 (100.00 %)	0 (0.00%)	0 (0.00%)			GG vs. GA + AA	4.09×10^{-92}	3.00×10^{-91}	0.00 (0.00–0.00)	0	0	0.00 (0.00–0.00)
rs1051790 (C/G)	C	CC	CG	GG			Additive	0.0239	0.0292	1.95 (1.09–3.48)	0.0187	0.0257	2.02 (1.12–3.62)
B27 positive	1507 (96.11%)	723 (92.22%)	61 (7.78%)	0 (0.00%)	0.031	0.0426	CC + CG vs. GG	0	0	Inf (Inf– Inf)	0	0	Inf (Inf– Inf)
B27 negative	206 (92.79%)	96 (86.49%)	14 (12.61%)	1 (0.90%)			CC vs. CG + GG	0.0454	0.0555	1.85 (1.01–3.39)	0.0374	0.0514	1.91 (1.04–3.51)
rs41539919 (A/T)	T	AA	AT	TT			Additive	1.0000	1.0000	Inf (0.00– Inf)	1.0000	1.0000	Inf (0.00– Inf)
B27 positive	40 (2.55%)	745 (95.03%)	38 (4.85%)	1 (0.13%)	0.0267	0.0392	TT + ATvs. AA	1.0000	1.0000	Inf (0.00– Inf)	1.0000	1.0000	Inf (0.00– Inf)
B27 negative	0 (0.00%)	111 (100.00 %)	0 (0.00%)	0 (0.00%)			TT vs. AT + AA	0	0	Inf (Inf– Inf)	0	0	Inf (Inf– Inf)
rs1051792 (G/A)	G	GG	GA	AA			Additive	7.52×10^{-13}	2.36×10^{-12}	3.76 (2.62–5.40)	5.49×10^{-13}	2.41×10^{-12}	3.85 (2.67–5.55)
B27 positive	1299 (82.84%)	521 (66.45%)	257 (32.78%)	6 (0.77%)	<0.0001	<0.0001	GG + GA vs. AA	1.77×10^{-8}	3.74×10^{-8}	17.20 (6.39–46.29)	1.86×10^{-8}	2.73×10^{-8}	17.92 (6.56–48.99)
B27 negative	138 (62.16%)	40 (36.04%)	58 (52.25%)	13 (11.71%)			GG vs. GA + AA	2.85×10^{-9}	8.62×10^{-9}	3.52 (2.32–5.32)	1.80×10^{-9}	6.71×10^{-9}	3.61 (2.38–5.49)
rs3819268 (A/T)	A	AA	AT	TT			Additive	0.3379	0.3540	1.40 (0.70–2.81)	0.2713	0.3141	1.48 (0.74–2.98)
B27 positive	1517 (96.75%)	733 (93.49%)	51 (6.51%)	0 (0.00%)	0.4278	0.4953	AA + AT vs. TT	1.0000	1.0000	Inf (0.00– Inf)	0	0	Inf (Inf– Inf)
B27 negative	212 (95.50%)	102 (91.89%)	8 (7.21%)	1 (0.90%)			AA vs. AT + TT	0.5282	0.5534	1.27 (0.61–2.65)	0.4467	0.5172	1.33 (0.63–2.81)
rs1051794 (G/A)	G	GG	GA	AA			Additive	2.61×10^{-12}	5.74×10^{-12}	3.56 (2.50–5.09)	1.63×10^{-12}	4.49×10^{-12}	3.66 (2.56–5.26)
B27 positive	1293 (82.46%)	517 (65.94%)	259 (33.04%)	8 (1.02%)	<0.0001	<0.0001	GG + GA vs. AA	3.20×10^{-8}	6.08×10^{-8}	12.87 (5.20–31.82)	3.00×10^{-8}	4.13×10^{-8}	13.49 (5.37–33.85)
B27 negative	138 (62.16%)	40 (36.04%)	58 (52.25%)	13 (11.71%)			GG vs. GA + AA	5.37×10^{-9}	9.85×10^{-9}	3.44 (2.27–5.20)	3.16×10^{-9}	6.94×10^{-9}	3.54 (2.33–5.38)
rs1131896 (G/A)	A	GG	GA	AA			Additive	5.45×10^{-20}	3.00×10^{-19}	7.30 (4.77–11.16)	1.96×10^{-19}	2.16×10^{-18}	7.09 (4.63–10.86)
B27 positive	1013 (64.60%)	29 (3.70%)	497 (63.39%)	258 (32.91%)	<0.0001	<0.0001	AA + GA vs. GG	1.13×10^{-25}	6.23×10^{-25}	17.10 (10.05–29.08)	9.02×10^{-25}	9.92×10^{-24}	16.55 (9.69–28.26)
B27 negative	79 (35.59%)	44 (39.64%)	55 (49.55%)	12 (10.81%)			AA vs. GA + GG	9.08×10^{-6}	1.23×10^{-5}	4.05 (2.18–7.50)	7.64×10^{-6}	8.40×10^{-6}	4.12 (2.21–7.65)
rs17206680 (C/T)	C	CC	CT	TT			Additive	0.0207	0.0268	1.89 (1.10–3.24)	0.0157	0.0230	1.96 (1.14–3.37)
B27 positive	1492 (95.15%)	709 (90.43%)	74 (9.44%)	1 (0.13%)	0.0262	0.0392	CC + CT vs. TT	0	0	0.00 (0.00–0.00)	0	0	0.00 (0.00–0.00)

B27 negative	203 (91.44%)	92 (82.88%)	19 (17.12%)	0 (0.00%)			CC vs. CT + TT	0.0168	0.0217	1.95 (1.13–3.38)	0.0129	0.0189	2.02 (1.16–3.51)
rs1051796 (C/T)	C	CC	CT	TT			Additive	1.66×10^{-11}	2.80×10^{-11}	3.47 (2.42–4.99)	1.11×10^{-11}	2.21×10^{-11}	3.56 (2.47–5.14)
B27 positive	1290 (82.27%)	513 (65.43%)	264 (33.67%)	7 (0.89%)	<0.0001	<0.0001	CC + CT vs. TT	4.31×10−07	6.82×10−07	12.21 (4.63–32.22)	5.29×10−07	6.47×10−07	12.47 (4.65–33.44)
B27 negative	140 (63.06%)	40 (36.04%)	60 (54.05%)	11 (9.91%)			CC vs. CT + TT	9.99×10^{-9}	1.69×10^{-8}	3.36 (2.22–5.09)	5.47×10^{-9}	1.09×10^{-8}	3.47 (2.29–5.28)
rs1051797 (C/T)	C	CC	CT	TT			Additive	4.90×10^{-12}	9.79×10^{-12}	3.62 (2.52–5.22)	3.80×10^{-12}	9.29×10^{-12}	3.70 (2.56–5.36)
B27 positive	1297 (82.72%)	519 (66.20%)	259 (33.04%)	6 (0.77%)	<0.0001	<0.0001	CC + CT vs. TT	2.97×10^{-07}	5.12×10^{-07}	14.26 (5.16–39.41)	3.87×10^{-07}	5.01×10^{-07}	14.51 (5.16–40.76)
B27 negative	140 (63.06%)	40 (36.04%)	60 (54.05%)	11 (9.91%)			CC vs. CT + TT	3.92×10^{-9}	8.62×10^{-9}	3.48 (2.30–5.26)	2.39×10^{-9}	6.71×10^{-9}	3.58 (2.35–5.44)
rs1140700 (T/C)	T	TT	TC	CC			Additive	3.09×10^{-14}	1.13×10^{-13}	4.03 (2.81–5.77)	2.20×10^{-14}	1.21×10^{-13}	4.13 (2.87–5.94)
B27 positive	1243 (79.27%)	470 (59.95%)	303 (38.65%)	11 (1.40%)	<0.0001	<0.0001	TT + TC vs. CC	1.21×10^{-11}	3.83×10^{-11}	14.51 (6.70–31.45)	1.19×10^{-11}	2.19×10^{-11}	15.14 (6.90–33.21)
B27 negative	124 (55.86%)	32 (28.83%)	60 (54.05%)	19 (17.12%)			TT vs. TC + CC	3.83×10^{-9}	8.62×10^{-9}	3.70 (2.39–5.71)	2.46×10^{-9}	6.71×10^{-9}	3.79 (2.45–5.88)
rs1051799 (C/G)	C	CC	CG	GG			Additive	9.50×10^{-12}	1.74×10^{-11}	3.39 (2.39–4.82)	7.41×10^{-12}	1.63×10^{-11}	3.46 (2.43–4.94)
B27 positive	1303 (83.10%)	529 (67.47%)	245 (31.25%)	10 (1.28%)	<0.0001	<0.0001	CC + CG vs. GG	1.91×10^{-6}	2.79×10^{-6}	8.51 (3.53–20.55)	1.92×10^{-6}	2.22×10^{-6}	8.80 (3.60–21.55)
B27 negative	141 (63.51%)	41 (36.94%)	59 (53.15%)	11 (9.91%)			CC vs. CG + GG	2.02×10^{-9}	7.42×10^{-9}	3.54 (2.34–5.36)	1.47×10^{-9}	6.71×10^{-9}	3.62 (2.39–5.49)
rs41554616 (C/G)	C	CC	CG	GG			Additive	0.0002	0.0003	3.22 (1.74–5.93)	0.0002	0.0003	3.26 (1.76–6.04)
B27 positive	1531 (97.64%)	748 (95.41%)	35 (4.46%)	1 (0.13%)	0.0006	0.0010	CC + CG vs. GG	0	0	0.00 (0.00–0.00)	0	0	0.00 (0.00–0.00)
B27 negative	206 (92.79%)	95 (85.59%)	16 (14.41%)	0 (0.00%)			CC vs. CG + GG	8.88×10^{-5}	0.0001	3.50 (1.87–6.55)	9.27×10^{-5}	0.0002	3.54 (1.88–6.66)
rs1063635 (G/A)	G	GG	GA	AA			Additive	1.93×10^{-14}	8.51×10^{-14}	4.12 (2.87–5.92)	1.04×10^{-14}	7.61×10^{-14}	4.26 (2.95–6.15)
B27 positive	1234 (78.70%)	460 (58.67%)	314 (40.05%)	10 (1.28%)	<0.0001	<0.0001	GG + GA vs. AA	4.70×10^{-13}	1.79×10^{-12}	18.06 (8.25–39.56)	4.03×10^{-13}	8.05×10^{-13}	19.08 (8.60–42.33)
B27 negative	122 (54.95%)	32 (28.83%)	58 (52.25%)	21 (18.92%)			GG vs. GA + AA	1.55×10^{-8}	2.43×10^{-8}	3.51 (2.27–5.41)	7.93×10^{-9}	1.45×10^{-8}	3.64 (2.35–5.64)

* P_{Trend} indicates the *p*–value from the Cochran–Armitage trend test with 10,000 permutations. *MICA* cSNP rs1063630T>G, rs1063631T>G, rs1051786A>G, rs1051792G>A, rs1051794G>A, rs1131896G>A, rs1051796C>T, rs1051797C>T, rs1140700T>C, rs1051799C>T, rs1063635G>A were significantly associated with *HLA–B27* positivity in AS patients.

Table S6. Comparison of *MICA* alleles between AS patients and normal controls with HLA–B27 positive individuals.

MICA Allele	Estimated Frequency Trend Test			Logistic Regression		Logistic Regression Adjusted for Sex	
	AS (2N = 1568)	Control (2N = 82)	*p* value	P_{FDR} value	OR (95% CI)	P_{FDR} Value	OR (95% CI)
**MICA*019:01**	746 (47.58%)	29 (35.37%)	0.005	0.011	2.85 (1.52–5.35)	0.012	2.84 (1.51–5.33)

**MICA*008:01:01**	274 (17.47%)	22 (26.83%)	0.034	0.114	0.53 (0.30–0.92)	0.109	0.52 (0.30–0.91)
**MICA*010:01**	148 (9.44%)	7 (8.54%)	1	0.861	1.13 (0.49–2.58)	0.871	1.12 (0.49–2.57)
**MICA*002:01**	138 (8.80%)	8 (9.76%)	1	0.861	0.89 (0.41–1.93)	0.871	0.89 (0.41–1.93)
**MICA*004**	57 (3.64%)	5 (6.10%)	0.350	0.499	0.56 (0.21–1.49)	0.514	0.57 (0.21–1.51)
**MICA*012:01**	47 (3.00%)	6 (7.32%)	0.075	0.114	0.37 (0.15–0.93)	0.113	0.37 (0.15–0.93)
**MICA*045**	35 (2.23%)	1 (1.22%)	0.949	0.791	1.82 (0.25–13.20)	0.763	1.88 (0.26–13.63)
**MICA*033**	39 (2.49%)	1 (1.22%)	0.832	0.791	2.04 (0.28–14.83)	0.763	2.00 (0.27–14.51)
**MICA*007:01**	24 (1.53%)	0 (0.00%)	0.832	0.999	1(0.00–Inf)	0.999	1(0.00–Inf)
**MICA*018:01**	4 (0.26%)	1 (1.22%)	0.442	0.402	0.21 (0.02–1.88)	0.353	0.19 (0.02–1.74)
**others**	56 (3.57%)	2 (2.44%)					

Table S7. Comparison of *MICA* alleles between AS patients and normal controls with HLA−B27 negative individuals.

**MICA Allele**	**Estimated Frequency Trend Test**			**Logistic regression**		**Logistic Regression Adjusted for Sex**	
	**AS**(2N = 222)	**Control** (2N =1 676)	***p* Value**	**P_{FDR} Value**	**OR (95% CI)**	**P_{FDR} Value**	**OR (95% CI)**
**MICA*019:01**	19 (8.56%)	127 (7.58%)	0.687	0.864	1.14 (0.69 – 1.90)	0.925	1.11 (0.67 – 1.86)
**MICA*008:01:01**	58 (26.13%)	480 (28.64%)	0.437	0.864	0.88 (0.65 – 1.21)	0.917	0.86 (0.63 – 1.19)
**MICA*010:01**	44 (19.82%)	265 (15.81%)	0.121	0.402	1.34 (0.93–1.93)	0.392	1.34 (0.93–1.93)
**MICA*002:01**	52 (23.42%)	366 (21.84%)	0.585	0.864	1.09 (0.79–1.53)	0.925	1.09 (0.78–1.53)
**MICA*004**	16 (7.21%)	125 (7.46%)	0.893	0.996	0.97 (0.57–1.63)	0.999	1.03 (0.61–1.74)
**MICA*012:01**	8 (3.60%)	135 (8.05%)	0.019	0.122	0.44 (0.21–0.90)	0.145	0.45 (0.22–0.92)
**MICA*045**	16 (7.21%)	61 (3.64%)	0.025	0.122	2.04 (1.16–3.61)	0.083	2.17 (1.22–3.86)
**MICA*033**	0 (0.00%)	1 (0.06%)	0.025	0.999	0.00 (0.00–Inf)	0.999	0.00 (0.00–Inf)
**MICA*007:01**	2 (0.90%)	18 (1.07%)	1	0.996	0.84 (0.19–3.65)	0.925	0.78 (0.18–3.44)
**MICA*018:01**	1 (0.45%)	3 (0.18%)	0.786	0.864	2.53 (0.26–24.54)	0.925	1.96 (0.19–19.71)
**other**	6 (2.70%)	95 (5.67%)					
